# Application of an enhanced recovery after surgery pathway for distal pancreatectomy

**DOI:** 10.1093/bjsopen/zrac119

**Published:** 2022-10-12

**Authors:** Ghada Majid-Jarrar, Ismail Labgaa, Nermin Halkic, Nicolas Demartines, Martin Hübner, Didier Roulin

**Affiliations:** Department of Visceral Surgery, Lausanne University Hospital (CHUV), University of Lausanne (UNIL), Lausanne, Switzerland; Department of Visceral Surgery, Lausanne University Hospital (CHUV), University of Lausanne (UNIL), Lausanne, Switzerland; Department of Visceral Surgery, Lausanne University Hospital (CHUV), University of Lausanne (UNIL), Lausanne, Switzerland; Department of Visceral Surgery, Lausanne University Hospital (CHUV), University of Lausanne (UNIL), Lausanne, Switzerland; Department of Visceral Surgery, Lausanne University Hospital (CHUV), University of Lausanne (UNIL), Lausanne, Switzerland; Department of Visceral Surgery, Lausanne University Hospital (CHUV), University of Lausanne (UNIL), Lausanne, Switzerland

*Dear Editor*

Enhanced recovery after surgery (ERAS) pathways have shown safety and efficiency in reducing perioperative surgical stress and postoperative morbidity in various surgical fields. In pancreatic surgery the focus has always been on pancreato-duodenectomy (PD)^1^. The present study aimed to assess ERAS compliance for distal pancreatectomy (DP) especially in the postoperative interval, and to identify predictive factors of low compliance.

This is a retrospective analysis of all patients undergoing elective DP with or without *en bloc* splenectomy and enrolled in an ERAS programme in a tertiary referral centre from 1 October 2012 to 31 December 2018. Exclusion criteria were age under 18 years, emergency surgery, and lack of informed consent. Compliance with ERAS guidelines was assessed according to the recommended pathway (*Table S1*).

Eighty-three patients were included (*Table S2*). For each ERAS item, the compliance is detailed in *Fig. 1*. Overall, the mean compliance was 71 per cent. Pre- and intraoperative compliances were 99 per cent and 94 per cent respectively. Postoperative compliance was the lowest (48 per cent). The most challenging postoperative items to fulfil were early mobilization, balanced intravenous (i.v.) fluids administration, and urinary catheter removal on postoperative day (POD) 2. Overall compliance ≥ 65 per cent or higher was associated with a 5-day reduction in duration of hospital stay (*P* = 0.007), decreased rate of overall complications (53 *versus* 87 per cent, *P* = 0.002), and a lower comprehensive complication index (13.2 *versus* 27.7, *P* < 0.001). A multivariable regression analysis of predictive factors of postoperative outcomes identified overall compliance ≥ 65 per cent or higher as the only independent factor of reduced postoperative complications (HR 0.19, 95 per cent c.i. 0.06 to 0.62; *P* = 0.006) (*Table S3*). Multivariable models were run to identify predictors of compliance to the challenging items: blood loss was an important factor associated with a reduced likelihood to comply with i.v. fluid administration (HR 0.996, 95 per cent c.i. 0.993 to 0.998, *P* 0.001) and urinary catheter removal (HR 0.996, 95 per cent c.i. 0.994 to 0.999, *P* = 0.030). Laparoscopy increased the chances of urinary catheter removal on POD2 (HR 3.64, 95 per cent c.i. 1.20 to 11.07, *P* = 0.023), whereas no predictive factor was identified for early mobilization (*Table S4*).

**Fig. 1:**
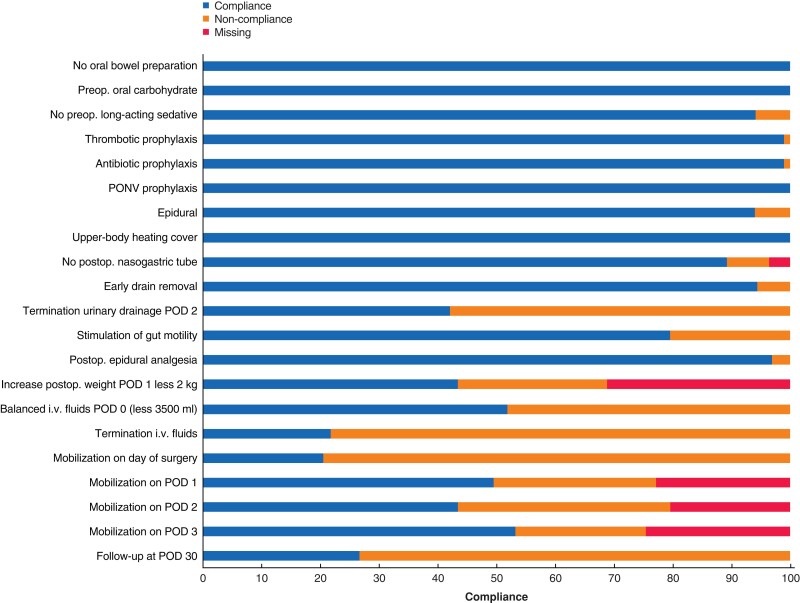
Perioperative compliance to enhanced recovery items PONV, postoperative nausea and vomiting; POD, postoperative day; i.v., intravenous.

As described in other studies on pancreatic surgery, compliance in the postoperative interval is one of the most challenging tasks, with compliance rates ranging from 30 to 70 per cent^2–4^. The postoperative compliance observed in the present study on DP was higher (48 per cent) than previously described for PD (30 per cent)^2^. Consistent with observations in other surgical fields^4^, the overall compliance had the greatest impact on optimal recovery and was the most significant predictor of improved outcome, with a significant reduction in complications after DP. Interestingly, increasing blood loss was identified as a predictive factor of failure of balanced i.v. fluids administration on the day of surgery. Reducing blood loss could potentially increase postoperative compliance. Laparoscopy was associated with decreased median blood loss in the DIPLOMA study^5^ and was also associated with early urinary catheter removal in this study. Thus, laparoscopy for DP should be recommended. Further data on larger multicentric cohort should be analysed to confirm these findings.

Compliance with the ERAS protocol for DP was independently associated with improved postoperative outcomes. Postoperative items such as early mobilization, balanced fluids administration, and urinary catheter removal represented the most challenging items. Increased blood loss was identified as an important determinant of compliance for i.v. fluid management and urinary catheter removal. These results emphasize the importance of compliance with the ERAS protocol and highlight its impact on recovery after DP.

## Supplementary Material

zrac119_Supplementary_Data

## Data sharing

Data are available upon request from the corresponding author.
