# Supplementary material for: Application of an enhanced recovery after surgery pathway for distal pancreatectomy
Source: BJS Open. 2022 Oct 12;6(5):zrac119. doi: 10.1093/bjsopen/zrac119 (PMC9553863; doi:10.1093/bjsopen/zrac119)
Supplement: zrac119_Supplementary_Data [file zrac119_supplementary_data.docx]

**Table S1** Definitions of enhanced recovery after surgery (ERAS) elements used for compliance calculation.

PONV, postoperative nausea and vomiting; POD, postoperative day; IV, intravenous

| **ERAS elements** | **Compliance definition** | |
| --- | --- | --- |
| Oral bowel preparation | | No bowel preparation |
| Preoperative oral carbohydrate | | Carbohydrate drink until 2 hours before surgery. |
| No preoperative long-acting sedative medication | | No preoperative long-acting sedatives on day of surgery |
| Thrombotic prophylaxis | | Preoperative low-molecular-weight heparin + sequential compression device |
| Antibiotic prophylaxis | | Antibiotic prophylaxis before skin incision |
| PONV prophylaxis | | PONV prophylaxis if two or more risk factors (female, non-smoker, previous PONV/motion sickness) |
| Epidural | | Thoracic epidural only for open surgery |
| Upper-body heating cover | | Use of upper-body forded-air heating cover intraoperatively |
| No postop nasogastric tube | | Removal of nasogastric tube before end of surgery |
| Early abdominal drain removal | | Early drain removal on POD3 if low risk |
| Termination urinary drainage POD 2 | | Transurethral catheter removal on POD 2 |
| Stimulation of gut motility | | Oral laxatives and/or chewing gum given postoperatively |
|  | |  |
| Increase weight POD 1 | | Increase of weight of less than 2kg |
| Balanced IV fluids POD 0 | | Less than 3500ml IV fluid on day of surgery |
| Termination IV fluids | | Termination of intravenous infusion less than 2 postoperative nights |
| Mobilisation on day of surgery | | Any mobilisation (to walk, to sit on a chair or rising from bed) |
| Mobilisation on POD 1 | | Mobilisation in total for more than 4 hours |
| Follow-up on POD 30 | | Clinical follow-up at POD 30 |

**Table S2** Patient’s characteristics and operative details.

| **Variables** | **N =83** | |
| --- | --- | --- |
| **Male gender (%)** | | 42 (51) |
| **Age, mean (SD), years** | | 61.8 (12) |
| **ASA score I-II (%)** | | 77 (93) |
| **BMI, mean (SD), kg/m^2^** | | 26.6 (5) |
| **Preoperative WHO status performance (%)** | |  |
| 0 | | 31 (37) |
| 1-3 | | 52 (63) |
| **Recent immunosuppressive treatment (%)** | | 3 (4) |
| **Neoadjuvant chemotherapy (%)**  **Comorbidities (%)** | | 1 (1) |
| Diabetes mellitus | | 15 (18) |
| Severe cardiac disease | | 4 (5) |
| Smoker | | 32 (39) |
| Alcohol usage | | 8 (10) |
|  | |  |
| **Procedure type (%)** | |  |
| Distal pancreatectomy with splenectomy | | 63 (76) |
| Spleen preserving distal pancreatectomy | | 20 (24) |
| **Surgical approach (%)** | |  |
| Open/converted | | 41 (49) |
| Laparoscopic | | 42 (51) |
| **Diagnosis (%)** | |  |
| Primary adenocarcinoma | | 47 (57) |
| Other primary malignancy | | 5 (6) |
| Metastasis or recurrence of any malignancy disease | | 4 (5) |
| Benign tumour/disease | | 21 (25) |
| Chronic pancreatitis | | 6 (7) |

BMI: body mass index, ASA: American society of anaesthesiology, WHO: world health organization.

**Table S3** Multivariable analysis of predictors of overall postoperative complications after distal pancreatectomy

| **Variables** | **Univariable** |  | **Multivariable** |  |
| --- | --- | --- | --- | --- |
|  | HR (95% CI) | p-value | HR (95% CI) | p-value |
| Age (years) | 0.97 (0.93-1.01) | 0.104 |  |  |
| Gender (female) | 0.87 (0.35-2.14) | 0.756 |  |  |
| ASA (III-IV) | 4.28 (0.89-20.49) | 0.069 | 3.55 (0.70-18.00) | 0.127 |
| BMI (kg/m^2^) | 1.05 (0.96-1.15) | 0.265 |  |  |
| Smoking | 0.69 (0.32-1.48) | 0.343 |  |  |
| En-bloc splenectomy | 1.10 (0.78-1.56) | 0.587 |  |  |
| Laparoscopy | 1.47 (0.63-3.43) | 0.369 |  |  |
| Blood loss (ml) | 1.00 (1.00-1.00) | 0.406 |  |  |
| Compliance >65% | 0.17 (0.05-0.56) | 0.004 | 0.19 (0.06-0.62) | 0.006 |

HR: hazard ratio; CI: confidence interval; ASA: American Society of Anesthesiologist; BMI: body mass index.

**Table S4** Multivariable analysis of predictors of overall compliance and compliance to specific elements of enhanced recovery after distal pancreatectomy

| **Variables** | **Univariable** | |  | **Multivariable** |  |  |
| --- | --- | --- | --- | --- | --- | --- |
|  | HR | 95% CI | p-value | HR | 95% CI | p-value |
| **Mobilisation on POD 1** | | | | | | |
| Age (years) | 0.992 | 0.948-1.038 | 0.732 |  |  |  |
| Gender (female) | 0.792 | 0.285-2.202 | 0.654 |  |  |  |
| ASA (III-IV) | 0.617 | 0.166-2.301 | 0.472 |  |  |  |
| BMI (kg/m^2^) | 0.991 | 0.905-1.085 | 0.847 |  |  |  |
| Smoking | 1.269 | 0.497-3.241 | 0.619 |  |  |  |
| Associated -splenectomy | 1.107 | 0.757-1.618 | 0.600 |  |  |  |
| Laparoscopy | 0.833 | 0.288-2.413 | 0.737 |  |  |  |
| Blood loss (mL) | 0.999 | 0.997-1.000 | 0.164 |  |  |  |
| **Intravenous fluid less than 3500ml the first 24 hours** | | | | | | |
| Age (years) | 1.021 | 0.983-1.061 | 0,288 |  |  |  |
| Gender (female) | 1.400 | 0.589-3.325 | 0,446 |  |  |  |
| ASA (III-IV) | 1.900 | 0.609-5.932 | 0,269 |  |  |  |
| BMI (kg/m^2^) | 0.979 | 0.902-1.063 | 0,614 |  |  |  |
| Smoking | 1.012 | 0.484-2.115 | 0,975 |  |  |  |
| Associated -splenectomy | 0.794 | 0.564-1.117 | 0,185 |  |  |  |
| Laparoscopy | 1.510 | 0.679-3.358 | 0,312 |  |  |  |
| Blood loss (mL) | 0.996 | 0.993-0.998 | <0.001 | 0.996 | 0.993-0.998 | **<0.001** |
| **Urinary catheter removal on POD 2** | | | | | | |
| Age (years) | 0.988 | 0.951-1.026 | 0.521 |  |  |  |
| Gender (female) | 1.043 | 0.432-2.522 | 0.925 |  |  |  |
| ASA (III-IV) | 0.905 | 0.289-2.836 | 0.864 |  |  |  |
| BMI (kg/m^2^) | 0.955 | 0.876-1.043 | 0.307 |  |  |  |
| Smoking | 0.664 | 0.304-1.452 | 0.305 |  |  |  |
| Associated -splenectomy | 0.577 | 0.393-0.849 | 0.005 | 0.644 | 0.409-1.015 | 0.058 |
| Laparoscopy | 4.700 | 1.742-12.677 | 0.002 | 3.640 | 1.197-11.068 | **0.023** |
| Blood loss (mL) | 0.996 | 0.994-0.998 | 0.001 | 0.996 | 0.994-0.999 | **0.003** |

HR: hazard ratio; CI: confidence interval; POD: postoperative day; ASA: American Society of Anesthesiologist; BMI: Body Mass Index.
